# Presacral myelolipoma, case report and literature review

**DOI:** 10.1016/j.amsu.2020.07.002

**Published:** 2020-08-02

**Authors:** Andriandi, A.H.G. Cleven, D.F. Hanff, H. Hartgrink, P.D.S. Dijkstra

**Affiliations:** a Department of Orthopaedic and Traumatology, Adam Malik General Hospital / Faculty of Medicine Universitas Sumatera Utara, Medan, Indonesia; b Department of Pathology, Leiden University Medical Center, Leiden, the Netherlands; c Department of Radiology, Leiden University Medical Center, Leiden, the Netherlands; d Department of Surgery Oncology, Leiden University Medical Center, Leiden, the Netherlands; e Department of Orthopedics, Leiden University Medical Center, Leiden, the Netherlands

**Keywords:** Myelolipoma, Extra-adrenal myelolipoma, Presacral myelolipoma

## Abstract

**Introduction:**

Myelolipomas are very rare benign tumours consisting of hematopoietic cells and mature adipose tissues. They are most commonly found in the adrenal glands. However, there have been several reported cases of extra-adrenal myelolipomas, most commonly in the presacral region. Nearly all presacral lesions are small and asymptomatic; thus, most are discovered incidentally on imaging studies.

**Presentation of case:**

We report two cases of presacral myelolipomas. The first is a 48-year-old female presenting with atypical back pain, found to have a mass in her presacral region with a size of 3,3 cm. The second case is a 59-year-old female, who presented for evaluation of a hip fracture, found to have a 4,7 cm presacral lesion. Both presacral myelolipomas were discovered incidentally and were confirmed by percutaneous guided fine-needle aspiration biopsy. Both were treated conservatively.

**Discussion:**

Accepted indications for the surgical excision of myelolipomas are symptomatic tumour, size >4 cm, metabolically active tumour, and a suspicion of malignancy on an imaging study. However, previous reports have documented that nearly half of the conservatively managed myelolipomas with a mean initial size of 5,1 cm, has increased in size or became symptomatic over a 3-years period.

**Conclusion:**

We conclude that symptomatic presacral myelolipomas or lesions larger than 4 cm should be en-bloc resected, and we present an intuitive decision-making algorithm.

## Highlight

• Presacral myelolipoma is an orphan disease.
• We emphasize the role of imaging in the differential diagnosis of presacral tumours.
• Percutaneous biopsy is rarely indicated to distinguish the diagnosis.
• We recommend enbloc resection for lesion larger than 4 cm.
• We present an intuitive decision-making algorithm.

## 1 Introduction

Myelolipomas are rare benign lesions containing mature adipose cells and a combination of myeloid and erythroid elements [1]. The incidence at autopsy ranges from 0.08% to 0.4% [2]. In general, it is an incidental finding with an expected-increase of incidence rate, due to the advances in imaging techniques [3,4].

Myelolipomas are found most commonly in the adrenal glands, there have been at least 43 reported cases of extra-adrenal myelolipomas, about more than half of which occurred in a presacral location [5]. There are nearly 40 cases of presacral myelolipomas reported in the English literature [6]. They have also been found in the mediastinum, lungs, liver or stomach [3,7]. They typically are discovered on patients of ages 50–60 years old and are more predominant in females [1,8]. The first extra-adrenal myelolipoma case was described in 1933 by Blaisdell et al. where it occurred in the presacral region. They presented a case of extra-medullary hematopoiesis in retroperitoneal tumour with a presacral mass of size 11 × 9x6.5 cm in a 64 years old female, which was later en-bloc resected.

Though myelolipomas are in general hormonally inactive, some reports were linking them with Conn's syndrome, Cushing syndrome or adrenal hyperplasia [8,9]. We describe the clinical course of two patients with extra-adrenal myelolipomas located in the presacral region.

## 2 Case report

**Fig. 1:**
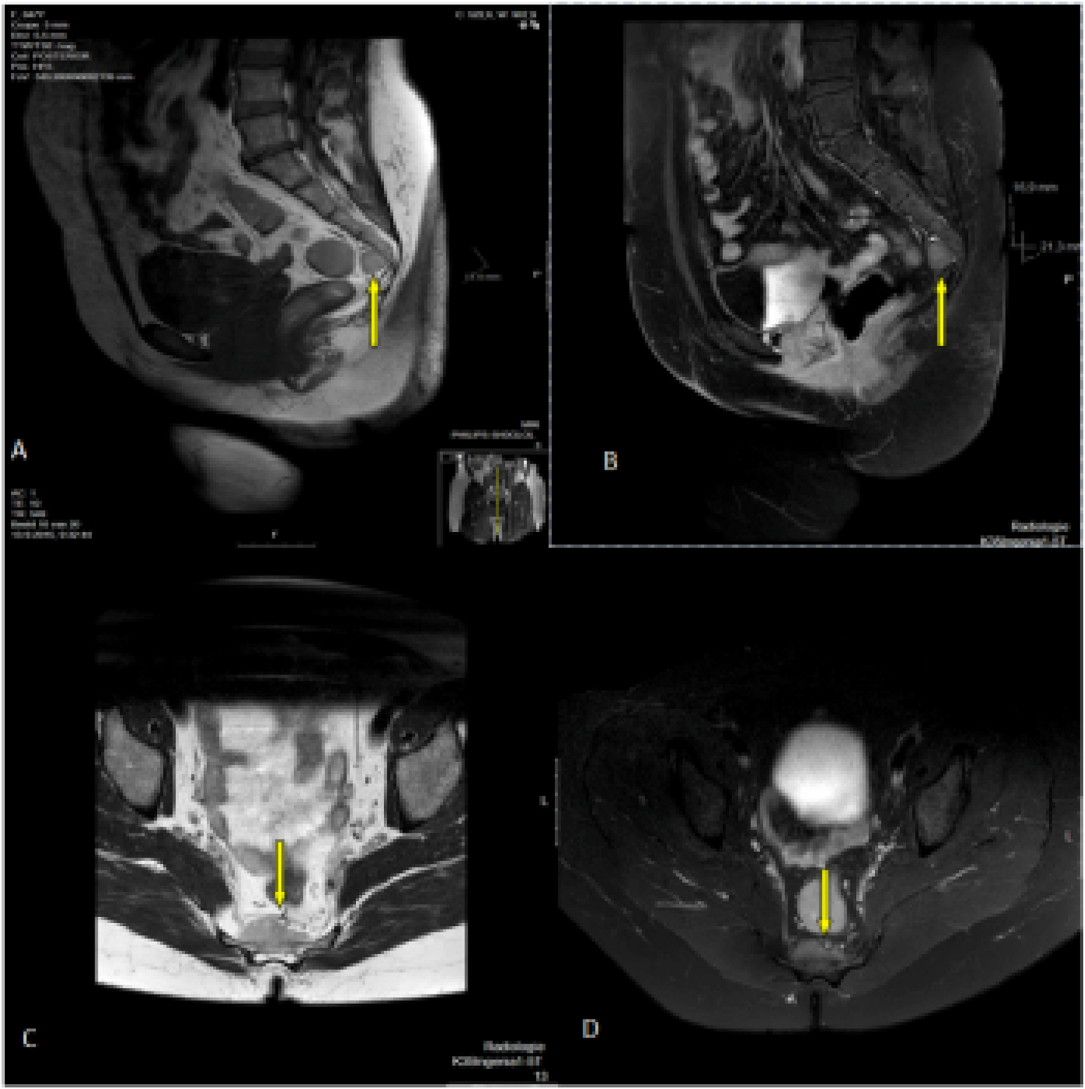
(A and B) Sagittal MRI scans showing how the lesions are in contact with the sacrum where the radiological characteristics of the lesions can be observed; (C and D) Coronal MRI scans showing the lobulated pelvic mass located immediately anterior to the sacrum with no invasion of surrounding structures (arrow).

The first case is a 48-year-old female who visited our outpatient clinic with atypical low back pain. On physical examination, there were normal sensations in the buttock and groin area without any neurological deficit. The range of motion of the lumbar spine and hip is without impairment. Conventional radiographs showed no abnormalities. Magnetic resonance imaging presented mild degenerative changes of the lumbar spine and a presacral soft tissue tumour at the level of S4–S5, sharp and clearly well defined, exophytic with a broad base anteriorly of the sacrum and sized 1,8 × 3,3 × 1,8 cm (Fig. 1). The sacral bone and the mesorectal fascia are without any apparent destruction or tumour infiltration. Later, a biopsy confirmed the diagnosis of myelolipoma (Fig. 3). On the last follow up, at 6 months after diagnosis, the patient has no complaints and is without any symptoms of local recurrence.

**Fig. 2:**
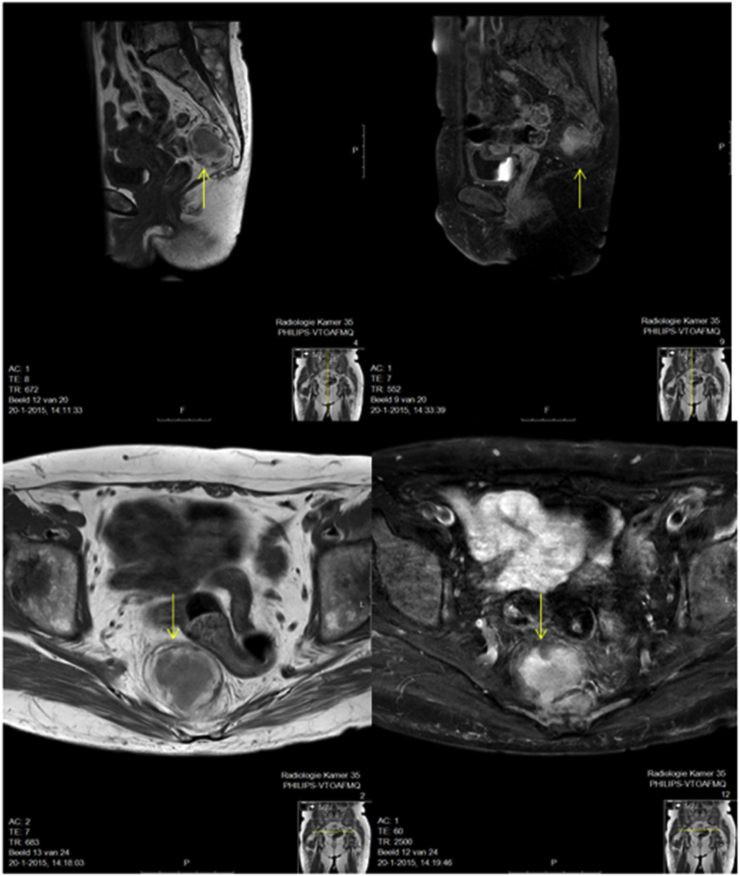
(A and B) Sagittal MRI scans showing how the lesions are in contact with the sacrum; (C and D) Coronal MRI scans showing the lobulated pelvic mass located immediately anterior to the sacrum with no invasion of surrounding structures (arrow).

The second case is a 59-year-old female who visited our outpatient clinic for an evaluation of a femoral neck fracture. There were no complaints of back pain. A CT scan was performed to evaluate the hip fracture, which incidentally presented a presacral lesion, partly soft tissue and noted with some fat content. MRI showed a mass on the anterior side of the level sacral S5 and coccyges, adjacent to the cortex but without bone destruction. The mass of size 4,2 × 4,2 × 4,7 cm is partly composed of fat with a solid, homogenous central component. Neither the sacral nerve roots nor the spinal canal was involved. (Fig. 2).

**Fig. 3:**
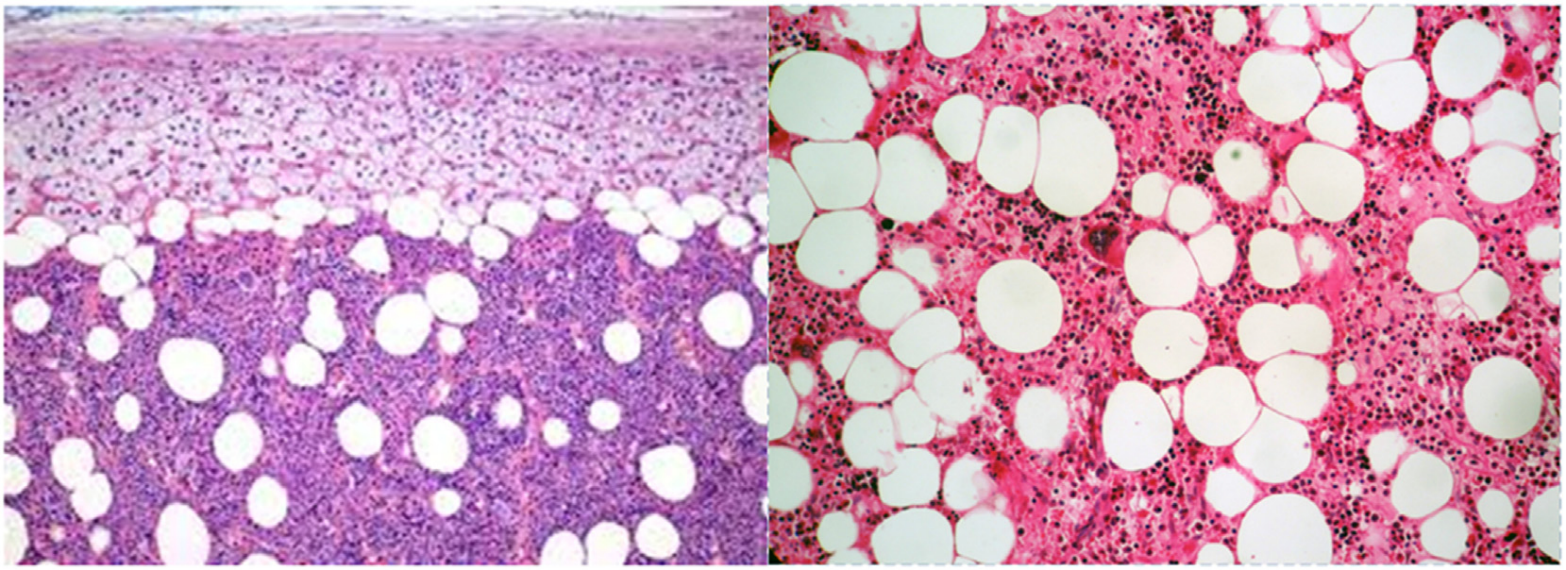
Photomicrograph from the biopsy specimen using H&E (hematoxycilin and eosin) staining. Image showed mature adipose tissue with prominent cellular stroma. The images also showed that the stroma consisted of all three hematopoietic cell lineages; myeloid, erythroid, and megakaryocytic forming cell lines.

A CT-guided biopsy was performed, and it revealed the histology of the specimen as mature adipose tissues with tri-lineage hematopoietic elements without any evidence of dysplasia, thus confirming the diagnosis of myelolipoma. (Fig. 3). The patient preferred conservative treatment. On the latest MRI, 6 months after the first diagnosis, no tumour progression was found.

## 3 Methodology

The systematic review of the literature used the search keywords “Presacral Myelolipoma” in PubMed, resulting in 32 articles published in English (Table 1) with a total of 39 reported cases. Table 1 lists all of the reported cases, with the earliest publication year of 1933, describing the first known case of presacral myelolipoma.

**Table 1:** Summary of all studies identified on Presacral Myelipoma.

AUTHOR	SYMPTOMS	SEX	AGE	SIZED	TREATMENT	IMAGING
Lee JJ et al. (2016)	Abdominal pain	Female	69	Ф 7.6 cm	Not mentioned	CT Scan, MRI
	No symptoms	Female	81	Ф 11 cm		
	Urine retention	Female	67	Ф 4.9 cm		
	No symptoms	Female	80	Ф 5.2 cm		
	Bloating	Female	56	Ф 8.5 cm		
Arora K et al. [10] (2016)	Abdominal discomfort	Male	64	6 × 5 cm	Resection	CT Scan
Fourati H et al. [11] (2015)	Abdominal pain	Female	40	11,5 × 8,5 × 5 cm	Follow up	CT Scan, MRI
Varone V et al. [12] (2015)	No symptoms	Female	55	5 × 4 cm	Follow up	CT Scan, MRI
Gangliardo C et al. [13] (2014)	No symptoms	Female	74	Not mentioned	Resection	CT Scan, MRI
Leite M et al. [14] (2014)	No symptoms	Male	84	5,5 × 4 × 3 cm	Resection	CT Scan, MRI
Sagarra CE et al. (2014)	Abdomen discomfort	Male	74	4,5 × 3,2 cm	Resection	MRI
Itani M et al. [15] (2014)	No symptoms	Male	58	3.6 × 3.2 cm	Follow up	CT Scan
	Abdomen discomfort	Female	58	4.8 × 3.5 cm	Resection	CT Scan, MRI
Baker KS et al. [16] (2012)	No symptoms	Female	79	5,8 × 2,9 × 4,8 cm	Resection	CT Scan, MRI
Asuquo SE et al. [17] (2011)	No symptoms	Female	74	3.5 × 1.7 × 0.6 cm	Resection	CT Scan
Gill KR et al. [18] (2010)	Abdominal pain	Female	71	Not mentioned	Follow up	CT Scan, MRI
Hernandez AA et al. [19] (2008)	Abdominal pain	Female	64	8 × 6,5 cm	Resection	CT Scan
Gheith S et al. [20] (2008)	Abdominal pain	Female	85	12 × 10 × 6.5 cm	Resection	CT Scan
Liu YL et al. [21] (2008)	Abdominal discomfort	Female	65	12 × 9 × 5 cm	Resection	CT Scan
Dann PH et al. [22] (2008)	Abdominal pain	Female	82	4.5 × 3.5 cm	Resection	CT Scan, MRI
Skorpil M et al. [23] (2007)	No symptoms	Female	84	Not mentioned	Resection	MRI
Orsola A et al. [24] (2005)	No symptoms	Male	68	13 × 9 cm	Resection	CT Ssan
Gong Y et al. [25] (2005)	Non specific back pain	Female	83	3,5 cm	Not mentioned	CT Scan, MRI
Mariappan MR et al. [26] (2004)	No symptoms	Male	74	10 × 8 × 5.5 cm	Found on autopsy	Found on Autopsy
Giuliani A et al. [27] (2001)	No symptoms	Female	71	9 × 8 × 7 cm	Resection	USG, CT Scan, MRI
Saboorian MH et al. [28] (1999)	No symptoms	Female	84	8.5 cm	Follow up	MRI
Adetiloye VA et al. [29] (1996)	Constipation	Male	1,5	Not mentioned	Resection	USG
Prahlow JA et al. [30] (1995)	Urinary retention	Male	68	15 × 10 × 8 cm	Resection	MRI
Grignon DJ et al. [31] (1989)	Abdominal pain	Female	80	12 cm	Not mentioned	Not mentioned
	No symptoms	Females	68	7 cm	Found on autopsy	Found on autopsy
	No symptoms	Female	83	6 cm	Found on autopsy	Found on autopsy
Chan YF et al. [32] (1988)	Abdominal discomfort	Male	53	Not mentioned	Not mentioned	CT Scan
Massey GS et al. [33] (1987)	Urine retention	Female	60	15.5 × 14.5 × 14 cm	Resection	CT Scan, USG
Sutker B et al. [34] (1985)	No symptoms	Female	58	9 × 7,5 × 3 cm	Resection	CT Scan
Chen KT et al. [35] (1982)	No symptoms	Female	72	16 × 15 × 7 cm	Resection	Intravenous Pyelography
Fowler MR et al. [36] (1982)	Constipation	Female	70	5 cm	Resection	CT Scan
Labow SB et al. [37] (1977)	No symptoms	Female	47	Not mentioned	Follow up	Sigmoidoscopy
Dodge OG et al. [38] 1956)	Abdominal pain	Female	74	15 × 10 × 10 cm	Resection	Not mentioned
Blasidell et al. (1933)	Urinary Track Syndrome	Female	64	11 × 11 cm	Resection	Not mentioned

This work has been reported in line with the SCARE 2018 criteria [39].

## 4 Results

From the literature review, we discovered that the mean size of the lesions was 8,5 cm, and most of the cases (~70%) were treated with excision. Neither local recurrence nor specific complaint was recorded after excision without specific complaint.

According to the reviewed publications, most cases of presacral myelolipomas are reported in females (30/39 cases, 76.9%) with a sex-based ratio of around 4:1 with female predominance, and a median age of 68 (1,5–85 years old).

## 5 Discussion

Presacral myelolipomas are slow-growing benign tumours, with an incidence of 1: 40.000 [7,40]^.^ They are asymptomatic in 26–50% of the cases [40]. Presacral myelolipomas typically occurs in the older individuals between 50 and 70 years of age, with a female predominance of approximately 2:1 [41,42,43].

The youngest patient was reported by Adetiloye et al. (1996), which was a 1.5 years old boy with a history of urinary retention and constipation. The presacral mass was later successfully resected and confirmed to be a myelolipoma tumour. The oldest patient was an 85 years old female reported by Gheith et al. (2008). Clearly, the tumour can occur in individuals on a wide range of age. tumour.

The characteristic finding of a presacral myelolipoma (besides its location) is the presence of fatty tissues within the mass, which would appear lucent on conventional radiographs, hyperechoic on ultrasonographic images, but hypo-vascular on conventional angiograms [43,44]. However, the fatty tissues within a myelolipoma can only be definitively diagnosed with either a CT-scan or magnetic resonance imaging (MRI). A CT-scan would reveal a low attenuated tissue, while an MRI would reveal an increased signal-intensity at T1-weighted sequences and a decreased signal-intensity at fat-suppressed T1-weighted sequences [45,46]. In both our presented cases, there was no invasion to adjacent structures, (namely the bone or associated pelvic lymphadenopathies). Hematopoietic elements will reveal a low-to-intermediate signal intensity on T1-weighted images and intermediate-to-high signal on T2-weighted images [45]. Administration of gadolinium-based contrast agent may show enhancement of the soft-tissue elements [47]. We discovered similar masses on both patients: the mass was exposed on the side of the distal sacrum and coccyges, closely related to the cortex but without any destruction. The mass is partly composed of fat with a solid homogenous central component. No involvement of the neural structures or the spinal canal was noted. Imaging studies are accurate in diagnosing myelolipomas in up to 90% of the cases [1,3,48].

In general, the MRI is the modality of choice for the diagnosis and preoperative evaluation of retrorectal–presacral tumours. This is due to its ability to delineate peritumoural planes and to determine local invasion (sacral and/or rectal) and nerve involvement, with higher contrast resolution compared to CT-scan or endorectal ultrasound (ERUS) [49,50].

Fine-needle (image-guided) aspiration cytology can play a significant role in the diagnosis of such lesion if said lesion is approachable [3,7]. Gong et al. (2015) have shown that an accurate diagnosis can be established by fine-needle aspiration (FNA) [51]. The overall sensitivity and negative predictive values of the biopsy were 73% and 60%, respectively [52].

The differential diagnosis of fat-containing presacral masses should include several pathologies, including liposarcoma, teratoma, extra-medullary hematopoiesis and neurogenic tumours like chordomas or neurofibromas [9,3,53]. Both the clinical history and the imaging data may help to further exclude these differentials.

Myelolipomas are usually asymptomatic, thus diagnosed incidentally. However, in the particular cases of large-sized myelolipomas, symptoms may arise due to the mass effect on adjacent structures (i.e.the bladder, ureters, sacral nerve plexus, and rectum) [8,3,7]. In the presented literature review, most presacral myelolipomas will present with symptoms if the size is larger than 4 cm. The most common symptom is abdominal pain/discomfort. If the size is larger than 10 cm, the patient is at risk of urinary retention due to local compressions.

From the pathological point of view, presacral myelolipomas tend to be circumscribed by a thin fibrous pseudocapsule, as viewed in figure (C). The colour varies from yellow to pink-tan to red, determined by the amount of fat and erythrocytic components [54]. On histological examinations, tumours are composed of mature adipocytes and hematopoietic cells. The overall proportions of these constituents vary among and within tumours. Trilineage hematopoiesis, including erythroid cells, myeloid cells, and megakaryocytes, are interspersed among the adipocytes [55]. Areas of haemorrhage, dystrophic calcification, and lymphoid aggregates are often found in presacral myelolipomas [56]. Our patients’ pathology reported the presence of hematopoietic material interspersed with isolated adipocytes, all of which was suggestive of myelolipoma.

As myelolipomas are benign, the question is whether or not they should be resected. The mean size of the presacral myelolipoma in the 39 cases of reviewed literature was 8.5 cm (range of 3.5 cm–16 cm), with symptoms are often present in patients with a tumour size of more than 4 cm. In general, the smaller lesions (<4 cm) confirmed to be myelolipomas by cytology were managed by careful follow-up, and larger lesions (>4 cm) are often removed to avoid risk of spontaneous rupture and haemorrhage.

Varone et al. (2015) have reported a 55 years old female patient with a 5 × 4 cm presacral myelolipoma which were treated conservatively. Follow-up MRI at 5, 12 and 18 months of the presacral mass had shown lesion stability without significant interval changes in size, appearance, nor signal characteristics. Asuquo et al. (2011) have reported a 74 years old female with a presacral myelolipoma sized less than 4 cm treated with resection surgery. The patient w as resected due to a biopsy-confirmed diagnosis of myelolipoma, and that it was symptomatic. Sagarra et al. (2014) have reported another patient with a 4,5 × 3,2 cm lesion in the presacral area who were treated with surgery.This patient also received surgery due to being symptomatic and with a risk for haemorrhage. Larger presacral myelolipomas tend to become symptomatic as they compress of adjacent structures. Along with other symptoms, urinary retention, constipation, radiculopathy, sciatic pain, intralesional haemorrhage, and infarctions have been described.haemorr [3,7,56].

The AACE/AAES Guideline (2009) recommends that myelolipomas that are observed (not receiving surgical excision) should undergo radiological evaluation at 3 and 6 months continued by an annual interval for 1–2 years. Melck et al. [57] concluded that the cost to conduct the surveillance of myelolipomas for more than 9 years would exceed the cost of surgery. Imamura et al. has reported a tumour-size doubling-time of 16–31 months in a patient of bilateral myelolipoma [58].

Accepted indications for the surgical excision of myelolipomas are symptomatic tumour, size > 4 cm, metabolically active tumour, and a suspicion of malignancy on an imaging study (Grade C recommendation, EL 3) [[59], [60], [61]]. However, Han et al. reported that nearly half of the conservatively managed myelolipomas, with a mean initial size of 5,1 cm, has increased in size or became symptomatic over a 3-year period [62]. Malignant degeneration has not been documented. Only a few longitudinal studies on myelolipomas have been reported, with the largest series describing 16 myelolipomas, of which 13 cases were followed-up for a mean of 3.2 years (range 0.3–10.8) with serial CT and MR Imaging. Of those, six increased in size (46%), two decreased in size (15%), and five remained unchanged (38%) [62]. In presented cases, the tumour size is smaller than 5 cm and are asymptomatic, so we treated them conservatively with close MRI monitoring at 6 months, 12 months, and intended future surveillance at 2, 3, and 5 years.

**Fig. 4:**
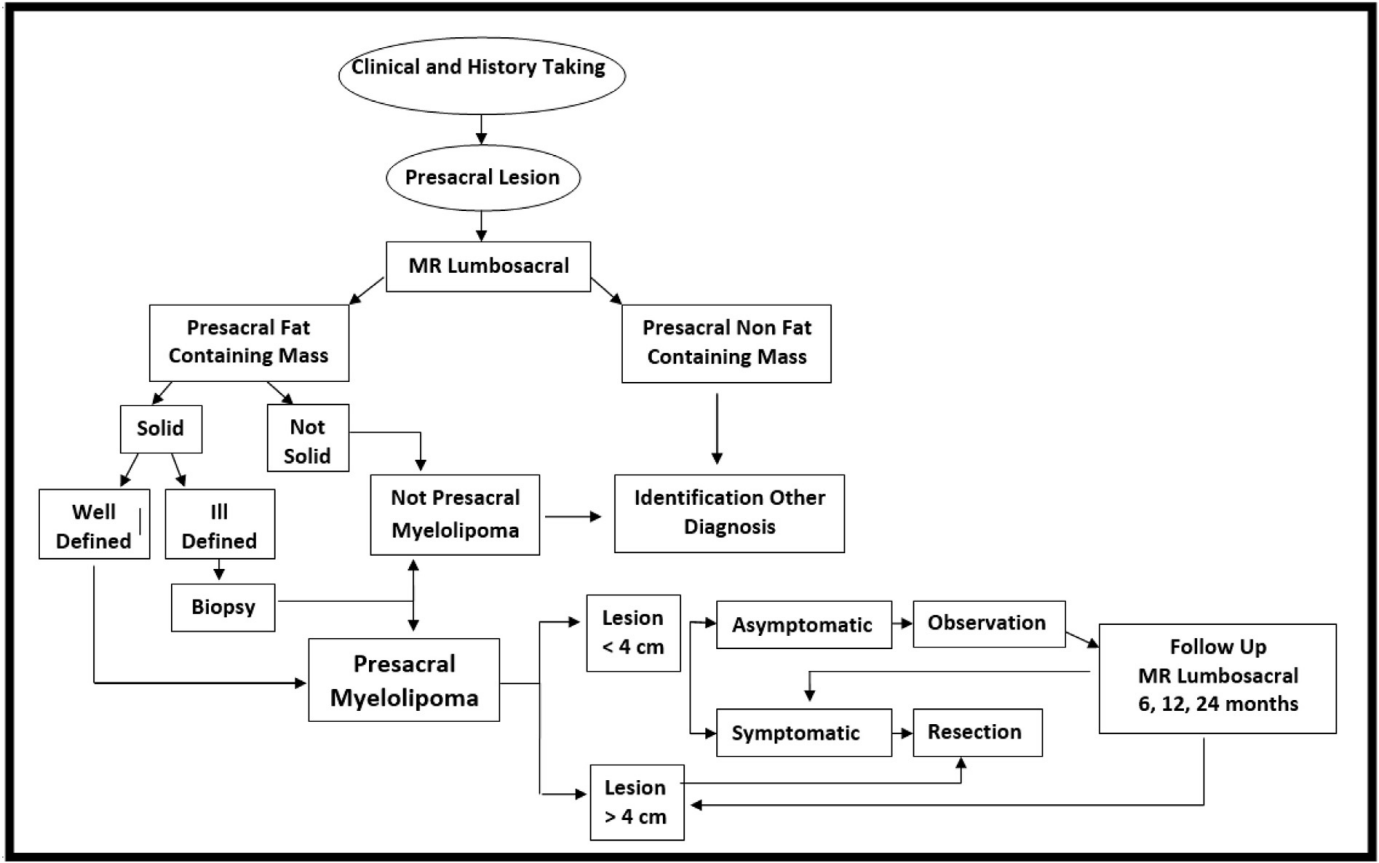
Proposed algorithm in the management of presacral myelolipoma.

Based on the literature review and our clinical expert opinion about presacral myelolipoma, we established an intuitive decision-making algorithm to guide the management (Fig. 4).

## 6 Conclusion

Presacral myelolipoma is an orphan disease. We report two cases of presacral myelolipoma, emphasizing the role of imaging in the differential diagnosis of presacral tumours. Two women presenting with an asymptomatic circumscribed presacral mass and based on MRI characteristics diagnosed of an extra-adrenal myelolipoma. The radiological presentation on gadolineum MRI is characteristic typical, percutaneous biopsy is rarely indicated to differentiate. We conclude that symptomatic presacral myelolipomas or lesions larger than 4 cm should be en-bloc resected, and we present an intuitive decision-making algorithm.

## Ethical approval

It was not required.

## Sources of funding

The authors declare that sponsors had no such involvement.

## Author contribution

A contributed to performed the operation, data collection, analysis and interpretation, manuscript drafting, revising, and approval for publishing; PDS Dijkstra contributed to performed the operation, data collection, analysis and interpretation, manuscript drafting, revising, and approval for publishing.

## Registration of research studies

1. Name of the registry: Andriandi Andriandi
2. Unique identifying number or registration ID: Researchregistry5767
3. Hyperlink to your specific registration (must be publicly accessible and will be checked): https://www.researchregistry.com/browse-the-registry#home/registrationdetails/5efa884c5445ba0015d22b27/

## Guarantor

Andriandi.

## Consent

Written informed consent was obtained from the patient for publication of this case report and accompanying images. A copy of the written consent is available for review by the Editor-in-Chief of this journal on request.

## Declaration of competing interest

The authors declare that there is no conflict of interests regarding the publication of this paper.
